# Sex differences in the efficacy of GLP‐1 receptor agonists: A systematic review and meta‐analysis of cardiovascular and renal outcome trials

**DOI:** 10.1111/dom.70123

**Published:** 2025-09-11

**Authors:** Hasan Fareed Siddiqui, Dua Ali, Maryam Sajid, Shaheer Qureshi, Hibah Siddiqui, Ali Hasan, David Ripley, Raheel Ahmed, Saad Ahmed Waqas

**Affiliations:** ^1^ Department of Medicine Dow University of Health Sciences Karachi Pakistan; ^2^ Department of Medicine National Heart and Lung Institute, Imperial College London London UK; ^3^ Department of Medicine Northumbria Hospitals NHS Foundation Trust Tyneside, Tyne and Wear UK; ^4^ Department of Medicine Newcastle University Newcastle upon Tyne UK; ^5^ Department of Medicine University of Sunderland Sunderland UK; ^6^ Department of Medicine Freeman Hospital Newcastle upon Tyne UK

**Keywords:** gender differences, GLP‐1 receptor agonists, hospitalization for heart failure, MACE, myocardial infarction, stroke

## Abstract

Sex‐based differences in the efficacy of glucagon‐like peptide‐1 receptor agonists (GLP‐1RAs) on cardiovascular, renal, and cerebrovascular outcomes remain unclear. This systematic review and meta‐analysis evaluated sex‐specific effects of GLP‐1RAs in patients with type 2 diabetes mellitus and related comorbidities. Randomised controlled trials and secondary analyses comparing GLP‐1RAs with placebo and reporting sex‐stratified data were included. Outcomes assessed included composite kidney outcomes, 3‐point major adverse cardiovascular events (MACE: cardiovascular death, non‐fatal myocardial infarction, or non‐fatal stroke), individual components of MACE, and hospitalization for heart failure (HHF). Hazard ratios (HRs) with 95% confidence intervals (CIs) were pooled using random‐effects models. Eleven trials comprising 85,273 patients (43, 339 receiving GLP‐1RAs; 41, 934 placebo) were analysed. GLP‐1RAs significantly reduced the risk of composite kidney outcomes by 20% in males (HR: 0.80; 95% CI: 0.69–0.92) and 31% in females (HR: 0.69; 95% CI: 0.54–0.87), with no significant sex interaction (*p* = 0.31). The risk of 3‐point MACE was reduced by 14% in males (HR: 0.86; 95% CI: 0.79–0.93) and 18% in females (HR: 0.82; 95% CI: 0.75–0.90; *p* = 0.47). Stroke risk decreased by 21% in males and 25% in females. No significant sex‐based differences were observed for cardiovascular death, myocardial infarction, or HHF. GLP‐1RAs reduce the risk of major cardiovascular, kidney, and cerebrovascular outcomes in both sexes, with consistent benefits across men and women. While variations between sexes were observed in certain outcomes, these differences did not reach statistical significance for interaction. Future trials should improve female representation and explore sex‐specific effects further.

## 1 INTRODUCTION

Type 2 diabetes mellitus (T2DM) is a well‐established risk factor for cardiovascular disease (CVD), yet emerging evidence indicates that its impact may differ between sexes. Women with T2DM appear to have a higher relative risk of adverse cardiovascular (CV) events compared to men, potentially due to biological, hormonal, and pharmacokinetic differences.^1^, ^2^ The 2022 American Diabetes Association (ADA) Standards of Medical Care recommend glucagon‐like peptide‐1 receptor agonists (GLP‐1RAs) and sodium‐glucose cotransporter‐2 inhibitors (SGLT2is) as first‐line therapies for individuals with T2DM who either have or are at high risk for atherosclerotic cardiovascular disease (ASCVD), heart failure (HF), or chronic kidney disease (CKD).^3^ Recent clinical trials have examined sex‐based differences in the effects of GLP‐1RAs on CV and renal outcomes. The FLOW trial (Research Study To See How Semaglutide Works Compared to Placebo in People With Type 2 Diabetes and Chronic Kidney Disease) reported a 21% reduction in composite kidney outcomes among females and a 30% reduction among males.^4^ Recent randomised controlled trials (RCTs) have reported mixed findings regarding sex‐specific CV benefits of GLP‐1RAs in reducing major adverse cardiovascular events (MACE). The AMPLITUDE‐O trial (Cardiovascular and Renal Outcomes with Efpeglenatide in Type 2 Diabetes) demonstrated a significant 44% reduction in MACE among females, suggesting potential cardioprotective benefits of GLP‐1RAs in women.^5^ In contrast, the SUSTAIN‐6 trial (Semaglutide and Cardiovascular Outcomes in Patients with Type 2 Diabetes) reported a significant reduction in MACE primarily among males by 32%, highlighting possible sex‐based differences in therapeutic response.^6^ These contrasting findings raise uncertainty about the extent to which these effects differ between men and women.

Despite the higher relative CVD risk in female T2DM patients, they remain underrepresented in RCTs, limiting the generalisability of findings. For instance, the SELECT and HARMONY trials included only 27.8% and 30% females, respectively.^7^, ^8^ Existing meta‐analysis has faced constraints due to the limited number of RCTs available, as well as the absence of data on renal outcomes and other key CVD indicators such as stroke. This raises concerns about statistical power and the applicability of current evidence to broader populations.^9^ To address these gaps, the present systematic review and meta‐analysis aim to synthesise the latest evidence on sex differences in the cardiovascular and renal effects of GLP‐1RAs, providing insights that may help inform precision medicine approaches in diabetes care.

## 2 METHODS

This systematic review and meta‐analysis was conducted in accordance with Preferred Reporting Items for Systematic Reviews and Meta‐Analyses (PRISMA) guidelines (PROSPERO REGISTRATION ID: CRD420251031670).^9^, ^10^ The literature search was conducted up to February 2025. Since the analysis was based on publicly available data, Institutional Review Board approval was not required.

### 2.1 Objectives, eligibility criteria, and outcomes

This meta‐analysis aimed to evaluate the impact of GLP‐1RAs on cardiovascular and renal outcomes across sex subgroups. Eligible studies met the following criteria: (1) RCTs or secondary analyses of RCTs; (2) comparison of GLP‐1RAs (subcutaneous or oral) with placebo; and (3) reporting of composite kidney outcome, 3‐point composite outcome of 3‐p MACE—consisting of CV death, non‐fatal myocardial infarction (MI), or non‐fatal stroke—CV death, stroke, MI, and hospitalisation for heart failure (HHF).

### 2.2 Data sources and search

A comprehensive literature search was performed across Medline, Scopus, and Cochrane Central from inception to the last week of February 2025, with no language restrictions. The search strategy incorporated a combination of relevant keywords and Medical Subject Headings (MeSH) terms, as detailed in Table S1. Additionally, the reference lists of selected studies and previous systematic reviews were manually examined to identify any relevant articles. To ensure thorough inclusion, clinical trial registries such as www.clinicaltrials.gov and proceedings from major cardiology conferences were also reviewed.^11^ The results from the systematic search were imported into the EndNote Reference Library, and duplicates were identified and removed. Two independent reviewers screened studies based on title and abstract, followed by a full‐text review to confirm eligibility (H.F.S and D.A.). Any disagreements were resolved by consulting a third reviewer (S.A.W.).

### 2.3 Data extraction and risk of bias assessment

A predesigned Excel spreadsheet was used to collect key study characteristics, baseline demographics, outcomes, and safety data. The Cochrane Risk of Bias tool was applied to assess study quality across seven domains, including sequence generation, allocation concealment, and blinding.^12^ Both data extraction and quality assessment were independently conducted by two reviewers (H.F.S. and D.A.), with any discrepancies resolved with the help of a third reviewer through discussion (S.A.W.).

### 2.4 Statistical Analysis

All statistical analyses were conducted using Review Manager version 5.3 (Cochrane Collaboration). Hazard ratios (HRs) with their corresponding 95% confidence intervals (CIs) were extracted from each trial for all outcomes. Study weights were determined using the generic inverse variance method, which accounts for study precision by incorporating the standard errors of the HRs.^14^ This method inherently considers both sample size and the number of events. To address potential heterogeneity among studies, a DerSimonian–Laird random‐effects model was applied. The generic inverse variance method enabled the integration of different effect measures, providing a more comprehensive analysis.^13^ Forest plots were generated to visually present the findings. Differences between sex subgroups were assessed using the chi‐square test, while heterogeneity across studies was evaluated using Higgins *I* ^2^, with an *I* ^2^ value of ≤50% considered acceptable. A *p*‐value of <0.05 was considered statistically significant. Additionally, leave‐one‐out sensitivity analyses were performed for each outcome.

## 3 RESULTS

The PRISMA flow chart (Figure S1) summarises the search and trial selection. From a total of 4739 initial results, 11 trials were found to meet the eligibility criteria.^4^, ^5^, ^6^, ^7^, ^14^, ^15^, ^16^, ^17^, ^18^, ^19^, ^20^ Secondary analyses of these trials that reported relevant subgroup data were also shortlisted.^21^, ^22^, ^23^, ^24^, ^25^, ^26^, ^27^, ^28^, ^29^, ^30^, ^31^ All included trials had a low risk of bias (Table S2). In total, 11 trials were included, which constituted 85,273 patients (*n* = 43, 339 receiving GLP‐1RAs; *n* = 41, 934 receiving placebo). Baseline characteristics of patients in each included trial are presented in Table 1.

**TABLE 1:** Baseline characteristics of patients in RCTs testing the efficacy of GLP1RAs across sexes.

Study	Select		Flow		Exscel		Leader		Rewind		Amplitude‐O		Harmony		Elixa		Sustain 6		Pioneer 6		Freedom‐Cvo	
Drug	Semaglutide 2.4 mg weekly		Semaglutide 1.0 mg weekly		Exenatide 2 mg weekly		Liraglutide 1.8 mg daily		Dulaglutide 1.5 mg weekly		Efpeglenatide 4 or 6 mg weekly		Albiglutide 30–50 mg weekly		Lixisenatide 10–20 mg weekly		Semaglutide 0.5–1 mg weekly		Semaglutide 14 mg oral daily		Exenatide 20–60 mg daily	
	Sema	Pla	Sema	Pla	Exen	Pla	Lira	Pla	Dula	Pla	Efpe	Pla	Albi	Pla	Lixi	Pla	Sema	Pla	Sema	Pla	Exen	Pla
Female (%)	27.8	27.5	29.4	31.1	38	38	11	12.4	46.6	46.1	34	30.8	30	31	30.4	30.9	40.1–27.0	41.5–38.5	31.9	31.4	37.5	35.9
Male (%)	72.2	72.5	70.6	68.9	62	62	14.1	16.2	53.4	53.9	66	69.2	70	69	69.6	69.1	59.9–63.0	58.5–61.5	68.1	68.6	62.5	64.1
Mean Age (years)	61.6	61.6	66.6	66.7	62	62	64.2	64.4	66.2	66.2	64.6	64.4	32.3	32.3	59.9	60.6	64.6–64.7	64.8–64.4	66	66	63	63
Mean BMI (kg/m^2^)	33.3	33.4	31.9	32	31.8	31.7	32.5	32.5	32.3	32.3	32.9	32.4	‐	‐	30.1	30.2	‐	‐	32.3	32.3	32.4	31.9
HF overall, *n* (%)	2155 (24.5)	2131 (24.2)	342 (19.4)	336 (19.0)	1161 (15.8)	1228 (16.6)	‐	‐	421 (8.5%)	432 (8.7%)	487 (17.9)	250 (18.4)	954 (20%)	968 (20%)	682 (22.5)	676 (22.3)	201 (24.3)‐ 180 (21.9)	190 (23.1)‐206 (25.0)	‐	‐	325 (15.7)	343 (16.5)
HFpEF, *n* (%)	1174 (13)	1099 (12)	167 (9)	158 (9)	499 (7)	561 (8)	‐	‐	‐	‐	‐	‐	‐	‐	‐	‐	‐	‐	‐	‐	‐	‐
HFrEF, *n* (%)	654 (7)	693 (8)	62 (4)	61 (3)	169 (2)	128 (2)	‐	‐	‐	‐	‐	‐	‐	‐	‐	‐	‐	‐	‐	‐	‐	‐
Systolic BP	131	130.9	138.9	138.4	‐	‐	135.9	135.9	137.1	137.3	135.1	134.4	134.8	134.7	129	130	‐	‐	135	136	137.5	137.5
Diastolic BP	79.4	79.2	76.8	76.1	‐	‐	77.2	77	78.4	78.5	76.8	76.6	76.8	76.8	‐	‐	‐	‐	76	76	79.5	80
T2DM, *n* (%)	0	0	1767 (100)	1766 (100)	7356 (100)	7296 (100)	4668 (100)	4672 (100)	4949 (100)	4952 (100)	2717 (100)	1359 (100)	4731 (100)	4732 (100)	3034 (100)	3034 (100)	1648 (100)	1649 (100)	1591 (100)	1592 (100)	2075 (100)	2081 (100)
CKD*, *n* (%)	970 (11)	938 (11)	1767 (100)	1766 (100)	1565 (21)	1626 (22)	1116 (24)	1042 (22)	1081 (22)	1118 (23)	863 (31.8)	424 (31.2)	1098 (23)	1124 (24)	‐	‐	‐	‐	441 (28)	434 (27)	‐	‐
Mean eGFR (mL/min per 1.73 m^2^)	82.4	82.5	46.9	47.1	‐	‐	80.2	80.6	75.3	74.7	72.2	72.9	79.1	78.9	76.7	75.2	‐	‐	74	74	79	80

Abbreviations: Albi, Albiglutide; BMI, body mass index; BP, blood pressure; CKD, chronic kidney disease; Dula, dulaglutide; Efpe, Efpeglenatide; eGFR, estimated glomerular filtration rate; Exen, exenatide; HF, heart failure; HFpEF, heart failure with preserved ejection fraction; HFrEF, heart failure with reduced ejection fraction; Lira, liraglutide; Lixi, Lixisenatide; Pla, placebo; Sema, semaglutide; T2DM, type 2 diabetes mellitus; − means data not available.

### 3.1 Composite kidney outcome

**FIGURE 1:**
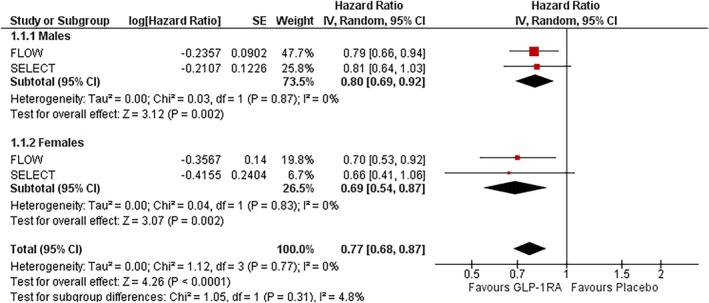
Forest plot illustrating the effect of GLP‐1RAs on composite kidney outcome, sub‐grouped by sex.

In males, GLP‐1RAs reduced the risk of composite kidney outcomes by 20% (HR: 0.80; 95% CI: 0.69–0.92; *I* ^2^ = 0%; *p* = 0.002). In females, the risk reduction was slightly greater at 31% (HR: 0.69; 95% CI: 0.54–0.87; *I* ^2^ = 0%; *p* = 0.002). There was no observed heterogeneity in either subgroup. The treatment effect did not significantly differ by sex (P‐interaction = 0.31) (Figure 1).

### 3.2 Major adverse cardiovascular events (MACE)

**FIGURE 2:**
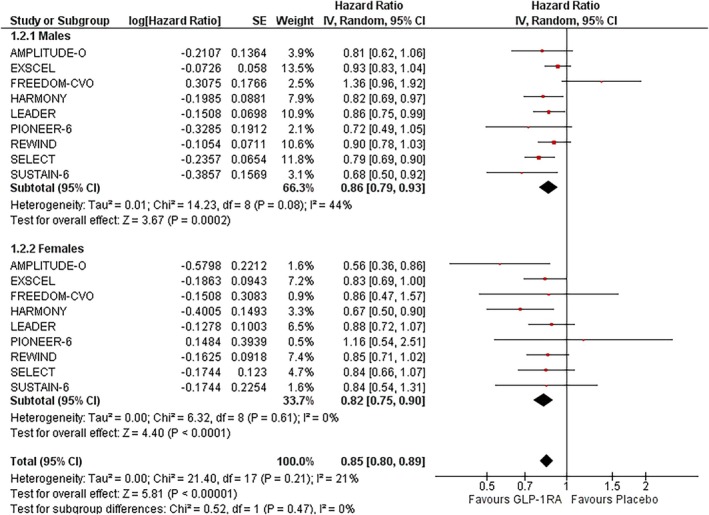
Forest plot illustrating the effect of GLP‐1RAs on MACE, sub‐grouped by sex.

In males, GLP‐1RAs reduced 3‐point MACE risk by 14% (HR: 0.86; 95% CI: 0.79–0.93; *I* ^2^ = 44%; *p* = 0.0002) (Figure 1). In females, the risk reduction was slightly greater at 18% (HR: 0.82; 95% CI: 0.75–0.90; *I* ^2^ = 0%; *p* <0.0001). There was no significant heterogeneity among female participants, whereas moderate heterogeneity was observed in males. Excluding the FREEDOM‐CVO trial in sensitivity analysis reduced heterogeneity while preserving the effect size in males (HR: 0.85; 95% CI: 0.80–0.90; *I* ^2^ = 0%; *p* <0.001) and females (HR: 0.82; 95% CI: 0.75–0.90; *I* ^2^ = 0%; *p* <0.001) (Figure S3). The treatment effect did not significantly differ by sex (P‐interaction = 0.47) (Figure 2).

### 3.3 Cardiovascular (CV) death

**FIGURE 3:**
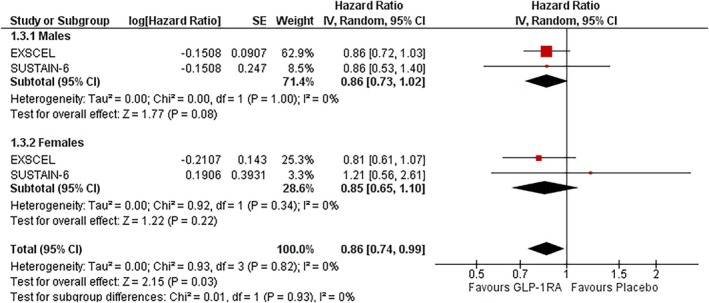
Forest plot illustrating the effect of GLP‐1RAs on CV death, sub‐grouped by sex.

In males, GLP‐1RAs were associated with a 14% reduction in CV death that did not reach statistical significance (HR: 0.86; 95% CI: 0.73–1.02; *I* ^2^ = 0%; *p* = 0.08) (Figure 1). In females, the effect size was similar (HR: 0.85; 95% CI: 0.65–1.10; *I* ^2^ = 0%; *p* = 0.22). There was no significant heterogeneity in either subgroup. The treatment effect did not significantly differ by sex (P‐interaction = 0.93) (Figure 3).

### 3.4 Myocardial infarction (MI)

**FIGURE 4:**
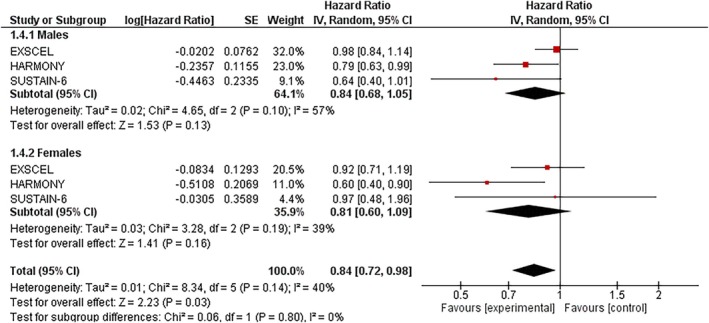
Forest plot illustrating the effect of GLP‐1RAs on MI, sub‐grouped by sex.

In males, GLP‐1RAs were associated with a nonsignificant 16% reduction in MI risk (HR: 0.84; 95% CI: 0.68–1.05; *I* ^2^ = 57%; *p* = 0.13). In females, the effect was similar but also nonsignificant (HR: 0.81; 95% CI: 0.60–1.09; *I* ^2^ = 39%; *p* = 0.16). Moderate heterogeneity was observed in males, while females showed lower variability. The treatment effect did not significantly differ by sex (P‐interaction = 0.80) (Figure 4).

### 3.5 Hospitalisation for HF

**FIGURE 5:**
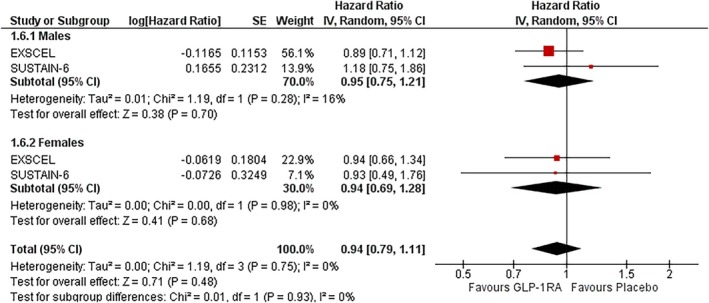
Forest plot illustrating the effect of GLP‐1RAs on HHF, sub‐grouped by sex.

In males, GLP‐1RAs showed a nonsignificant trend towards reducing HHF (HR: 0.95; 95% CI: 0.75–1.21; *I* ^2^ = 16%; *p* = 0.70). In females, the effect size was similar (HR: 0.94; 95% CI: 0.69–1.28; *I* ^2^ = 0%; *p* = 0.68). No significant heterogeneity was observed in either subgroup. The treatment effect did not significantly differ by sex (P‐interaction = 0.93) (Figure 5).

### 3.6 Stroke

**FIGURE 6:**
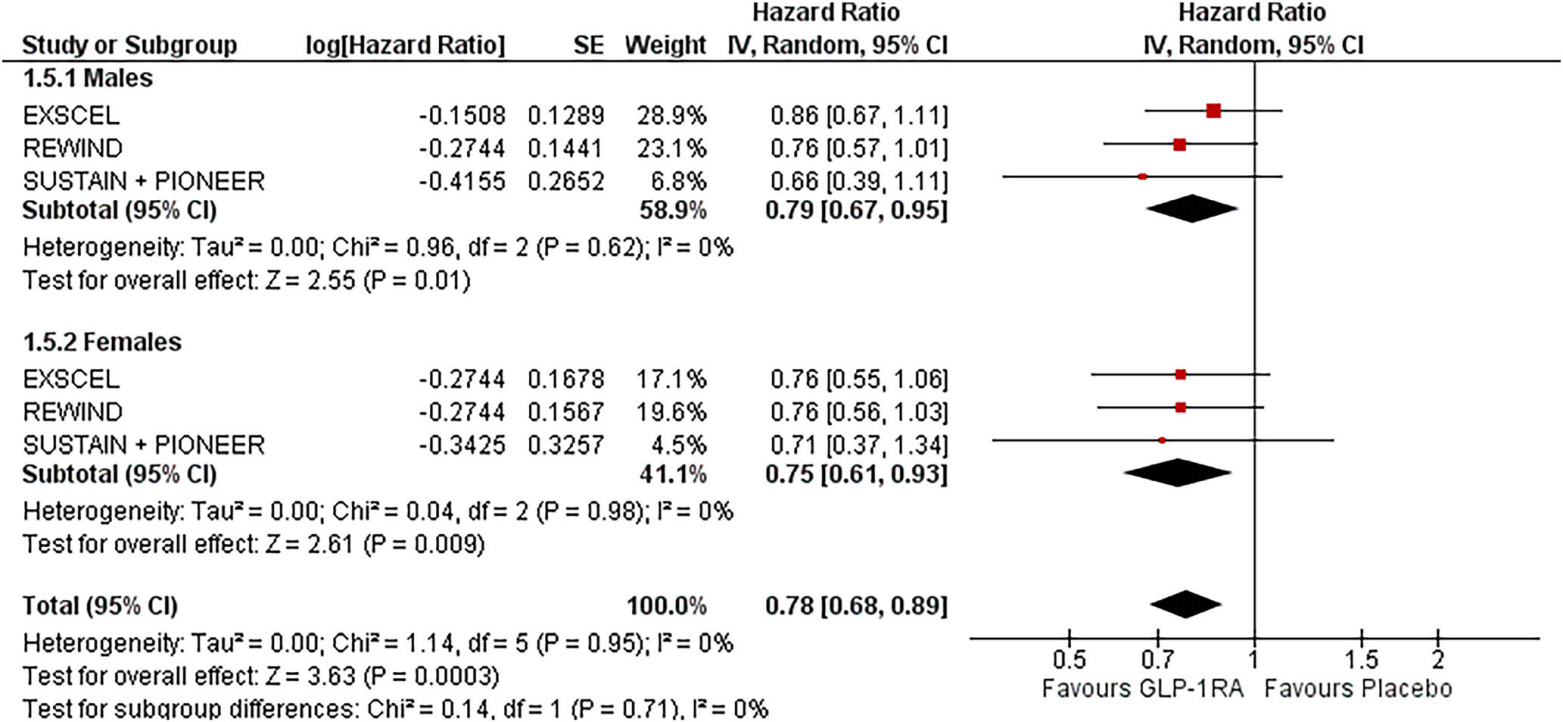
Forest plot illustrating the effect of GLP‐1RAs on stroke, sub‐grouped by sex.

In males, GLP‐1RAs significantly reduced stroke risk by 21% (HR: 0.79; 95% CI: 0.67–0.95; *I* ^2^ = 0%; *p* = 0.01) (Figure 1). In females, the risk reduction was greater at 25% (HR: 0.75; 95% CI: 0.61–0.93; *I* ^2^ = 0%; *p* = 0.009). Sensitivity analysis excluding the SUSTAIN 6 + PIONEER 6 pooled analysis yielded a significant reduction in males (HR: 0.81; 95% CI: 0.67–0.98; *I* ^2^ = 0%; *p* = 0.03) and females (HR: 0.76; 95% CI: 0.61–0.95; *I* ^2^ = 0%; *p* = 0.02) (Figure S4). There was no observed heterogeneity in either subgroup. The treatment effect did not significantly differ by sex (P‐interaction = 0.62) (Figure 6).

## 4 DISCUSSION

This meta‐analysis of 85, 273 patients identifies several important findings. First, GLP‐1RAs reduce the risk of MACE by 14% in men and 18% in women, with consistent benefits observed for stroke mortality indicating important protective effects for both sexes. While no individual trial demonstrated a statistically significant reduction in stroke mortality, this effect became apparent when the data were pooled (Figure 6). Second, GLP‐1RAs lowered the risk of the composite kidney outcome by 20% in men and 31% in women, with no evidence of treatment effect heterogeneity across the available data. However, GLP‐1RAs did not show significant benefits for CV death, MI, or HHF in both sexes. These outcomes were reported in a relatively limited number of studies, indicating that the absence of statistical significance may reflect insufficient statistical power rather than a genuine lack of effect.

GLP‐1RAs have demonstrated consistent cardiovascular and renal benefits across diverse populations.^32^, ^33^, ^34^, ^35^ These effects are attributed to multiple mechanisms, including attenuation of atherosclerotic progression, enhancement of endothelial function, promotion of weight loss, and suppression of pro‐inflammatory cytokines.^35^, ^36^, ^37^, ^38^, ^39^ These agents have also demonstrated efficacy in stroke prevention, with findings from the REWIND trial indicating that reductions in HbA1c accounted for 54% of the observed decrease in stroke risk, alongside a linear association between HbA1c improvement and stroke reduction across trials.^20^, ^40^ They also help improve renal outcomes by enhancing natriuresis and lowering glomerular hyperfiltration.^41^

Our findings are consistent with prior analyses; however, previous studies were based on smaller effect sizes and a limited number of outcomes, potentially restricting generalisability and precluding a comprehensive assessment.^9^ Moreover, a separate meta‐analysis investigating sex‐specific effects of GLP‐1RAs employed odds ratios, which do not account for event timing and may overestimate risk, rendering them less suitable for time‐to‐event analyses.^9^, ^43^ In contrast, the present study exclusively utilises HRs, offering a more precise and dynamic estimation of risk.

Large‐scale observational studies suggest that T2DM confers a 25%–50% greater excess risk of CVD in women compared to men.^44^ This disparity may, in part, be attributable to a greater degree of weight loss observed in women, potentially due to higher drug exposure related to lower average body weight, which may result in improved metabolic profiles and clinical outcomes.^45^ However, our study observed no significant sex‐specific differences in the cardiovascular impact of GLP‐1RAs. Notably, sex‐stratified analyses of SGLT2 inhibitors demonstrated a markedly low treatment effect in women compared to men.^46^ The consistent improvement in cardio‐renal outcomes across both sexes and specifically in women supports the broader clinical utility of GLP‐1RAs.

Heterogeneity in outcomes may be largely attributable to variations in trial design and patient demographics. Notably, follow‐up durations differed substantially across studies, ranging from 1.3 years in the FREEDOM CVO trial to 5.4 years in REWIND, potentially impacting the ability to detect long‐term CV events.^16^, ^20^ The short follow‐up in FREEDOM‐CVO may explain the observed heterogeneity for 3‐p MACE in our analysis.^16^ While most trials enrolled patients with T2DM and either established CVD, CKD or associated risk factors, some studies diverged from this inclusion framework. For example, the SELECT trial focused on obese individuals without diabetes; AMPLITUDE‐O included a proportion of patients receiving SGLT2 inhibitors, introducing the possibility of synergistic effects; and ELIXA specifically recruited patients with recent acute coronary syndrome.^5^, ^7^, ^14^

Biological differences in men and women have important implications for disease aetiology and manifestation, treatment response, and outcomes. In contrast to previous reports, our study also identified underrepresentation of women across RCTs, limiting the ability to fully characterise sex‐specific risks (Table 1).^47^ The roots of this disparity are complex. In the past, researchers primarily conducted studies on male participants, and the results were generalised to women, largely to avoid dealing with the challenges posed by hormonal variations in women and the potential unknown risks to pregnancy.^48^ This could also be attributed to women experiencing greater GLP‐1RA‐related adverse effects leading to early discontinuation. Existing evidence suggests that women may experience higher rates of gastrointestinal side effects, including nausea, vomiting, and GI bleeding.^49^, ^50^, ^51^ Women have a higher risk than men of being diagnosed with depression and experiencing suicidal thoughts or attempts following GLP‐1RA therapy.^52^ The lack of sex‐specific data may contribute to suboptimal outcomes, highlighting the need for inclusive research to improve care for both sexes.^53^ Future research should prioritise increased female representation in RCTs to better delineate sex‐specific differences, characterise adverse events, assess subgroups most likely to benefit, and establish evidence‐based criteria to guide clinical decision‐making. Moreover, real‐world data assessing adherence, discontinuation rates, and treatment durability will provide insights into the long‐term viability of GLP‐1RA therapy among men and women.

## 5 LIMITATIONS

Several limitations of this study should be considered. First, subgroup analyses were limited by the lack of a uniform population across the included trials, making it difficult to assess treatment effects in specific high‐risk groups. Although all trials were long‐term CVOTs, their primary inclusion criteria varied, adding to the heterogeneity. Second, while a random‐effects model was employed to account for heterogeneity, differences in trial design, GLP‐1RA type and dosage, as well as baseline patient characteristics, may affect the interpretation of results. Third, although GLP‐1RAs significantly reduced the risk of 3‐point MACE, myocardial infarction, stroke, hospitalisation for HF, CV death, and adverse events leading to treatment discontinuation, variations in follow‐up duration and patient demographics may limit the generalisability of these findings. Finally, due to insufficient data, a meta‐analysis of specific adverse events could not be performed, underscoring the need for future studies to comprehensively evaluate these outcomes.

## 6 CONCLUSION

This analysis provides substantial evidence that GLP‐1 RAs effectively reduce the risk of major clinical outcomes, including MACE, stroke, and composite kidney events, across both sexes, consistent with findings in the overall population. Additionally, GLP‐1 RAs demonstrated a beneficial impact on MI, CV death, and HHF. These findings underscore their therapeutic potential across sexes and highlight the need for further research to evaluate sex‐specific safety profiles and clinical applications.

## FUNDING INFORMATION

The authors received no funds, grants, or financial support for this study.

## CONFLICT OF INTEREST STATEMENT

The authors declare that they have no conflicts of interest.

## PEER REVIEW

The peer review history for this article is available at https://www.webofscience.com/api/gateway/wos/peer‐review/10.1111/dom.70123.

## Supporting information


**Table S1.** Detailed search string for extracted studies from databases.
**Table S2.** Risk of bias assessment of the included RCTs.
**Figure S1:** PRISMA flowchart
**Table S3.** Definitions of composite kidney outcomes in the included randomised controlled trials (RCTs).
**Figure S3.** Forest plot illustrating the effect of GLP‐1RAs on MACE after the exclusion of the FREEDOM‐CVO trial.
**Figure S4.** Forest plot illustrating the effect of GLP‐1RAs on stroke after the exclusion of the SUSTAIN + PIONEER‐6 trial.

## Data Availability

The data supporting the findings of this study were obtained from published Randomized Controlled Trials. The data and material used were publicly available.
