# Supplementary material for: Sex differences in the efficacy of GLP‐1 receptor agonists: A systematic review and meta‐analysis of cardiovascular and renal outcome trials
Source: Diabetes Obes Metab. 2025 Sep 11;27(12):6847–56. doi: 10.1111/dom.70123 (PMC12587241; doi:10.1111/dom.70123)

**Supplementary Appendix**

**Online supplementary material 1:** Search strategy, PRISMA flowchart and Risk of Bias Assessment

**Online supplementary material 2:** Sensitivity Analysis plots

**Online supplementary material 1:** Search strategy, PRISMA flowchart and Risk of Bias Assessment

**Online Table S1.** Detailed search string for extracted studies from databases.

| **Database** | **String** | **Results** |
| --- | --- | --- |
| **PubMed** | (("GLP-1 receptor agonist"[MeSH Terms] OR "GLP-1 receptor agonist"[All Fields] OR "glucagon-like peptide-1 receptor agonist"[All Fields] OR "liraglutide"[MeSH Terms] OR "liraglutide"[All Fields] OR "semaglutide"[MeSH Terms] OR "semaglutide"[All Fields] OR "dulaglutide"[MeSH Terms] OR "dulaglutide"[All Fields] OR "exenatide"[MeSH Terms] OR "exenatide"[All Fields] OR "albiglutide"[All Fields] OR "efpeglenatide"[All Fields])  AND  ("chronic kidney disease"[MeSH Terms] OR "chronic kidney disease"[All Fields] OR "renal failure"[MeSH Terms] OR "renal failure"[All Fields] OR "eGFR"[All Fields] OR "albuminuria"[MeSH Terms] OR "albuminuria"[All Fields] OR "kidney function"[All Fields] OR "nephropathy"[MeSH Terms] OR "nephropathy"[All Fields] OR "cardiovascular disease"[MeSH Terms] OR "cardiovascular disease"[All Fields] OR "MACE"[All Fields] OR "myocardial infarction"[MeSH Terms] OR "myocardial infarction"[All Fields] OR "stroke"[MeSH Terms] OR "stroke"[All Fields] OR "heart failure"[MeSH Terms] OR "heart failure"[All Fields])) | 1885 |
| **Cochrane Library** | ("GLP-1 receptor agonist" OR "glucagon-like peptide-1 receptor agonist" OR liraglutide OR semaglutide OR dulaglutide OR exenatide OR albiglutide OR efpeglenatide)  AND  ("chronic kidney disease" OR "renal failure" OR eGFR OR albuminuria OR "kidney function" OR nephropathy OR "cardiovascular disease" OR MACE OR "myocardial infarction" OR stroke OR "heart failure"):ti,ab,kw | 1322 |
| **Scopus** | TITLE-ABS-KEY ("GLP-1 receptor agonist" OR "glucagon-like peptide-1 receptor agonist" OR liraglutide OR semaglutide OR dulaglutide OR exenatide OR albiglutide OR efpeglenatide)  AND  TITLE-ABS-KEY ("chronic kidney disease" OR "renal failure" OR eGFR OR albuminuria OR "kidney function" OR nephropathy OR "cardiovascular disease" OR MACE OR "myocardial infarction" OR stroke OR "heart failure") | 1532 |

**Online Table S2.** Risk of bias assessment of the included RCTs.

| **Trial** | **Sequence generation** | **Allocation concealment** | **Blinding** | **Detection bias** | **Attrition bias** | **Other bias** |
| --- | --- | --- | --- | --- | --- | --- |
| **SELECT** | Low | Low | Low | Low | Low | Low |
| **FLOW** | Low | Low | Low | Low | Low | Low |
| **LEADER** | Low | Low | Low | Low | Low | Low |
| **REWIND** | Low | Low | Low | Low | Low | Low |
| **AMPLITUDE-O** | Low | Low | Low | Low | Low | Low |
| **HARMONY** | Low | Low | Low | Low | Low | Low |
| **ELIXA** | Low | Low | Low | Low | Low | Low |
| **SUSTAIN 6** | Low | Low | Low | Low | Low | Low |
| **PIONEER 6** | Low | Low | Low | Low | Low | Low |
| **FREEDOM-CVO** | Low | Low | Low | Low | Low | Unclear |

**Online Figure S1:** PRISMA flowchart

**Identification of studies via databases**

Records removed *before screening*:

Duplicate records removed
 (n = 756)

Records identified from: MEDLINE, Scopus, and Cochrane CENTRAL (n = 1586)

**Identification**

Records screened in total.

(n = 830)

Records excluded based on title and abstract.

(n = 678)

**Screening**

Records excluded.

Irrelevant studies (n=52)

Irrelevant outcome (n=23)

Review studies (n=19)

Inappropriate comparison= (n=36)

Protocols (n=8)

Ongoing RCT (n=3)

Potentially relevant articles identified for full text review
(n =152)

**Included**

Studies included in qualitative- synthesis (n = 11)

**Online Table S3.** Definitions of composite kidney outcomes in the included randomized controlled trials (RCTs).

| **Trial Name** | **Definition** |
| --- | --- |
| **SELECT** | Death from kidney disease, initiation of chronic kidney replacement therapy (dialysis or transplantation), onset of persistent eGFR <15 ml/min/1.73 m², persistent ≥50% reduction in eGFR from baseline, or onset of persistent macroalbuminuria |
| **REWIND** | New macroalbuminuria, sustained ≥30% decline in eGFR from baseline (confirmed by 2 measurements), or initiation of chronic renal replacement therapy |
| **FLOW** | First occurrence of kidney failure (initiation of dialysis, kidney transplantation, or sustained eGFR <15 ml/min/1.73 m² for ≥28 days), sustained ≥50% reduction in eGFR from baseline (for ≥28 days), or death from kidney-related or cardiovascular causes. |

**Online supplementary material 2:** Sensitivity Analysis plots

**Online Figure S3.** Forest plot illustrating the effect of GLP-1RAs on MACE after the exclusion of the FREEDOM-CVO trial.


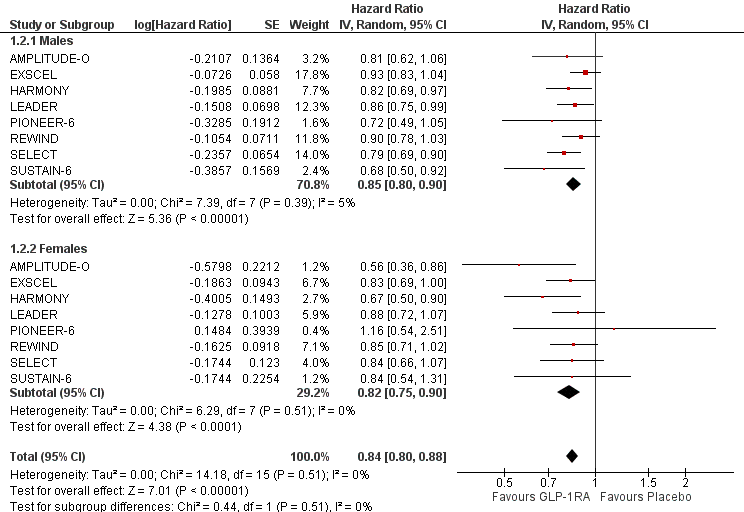


**Online Figure S4.** Forest plot illustrating the effect of GLP-1RAs on stroke after the exclusion of the SUSTAIN + PIONEER-6 trial.


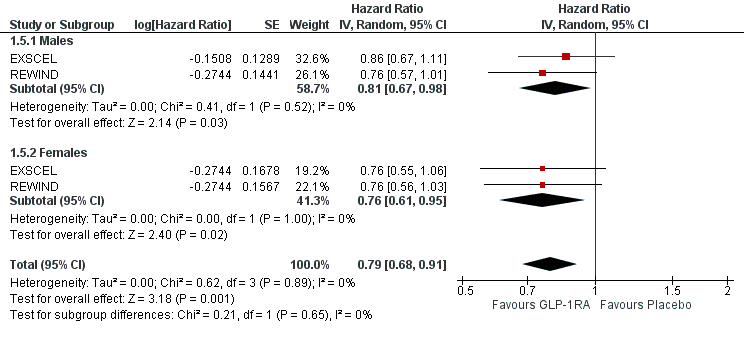

Supplement: Supplementary file 1 — Table S1. Detailed search string for extracted studies from databases. Table S2. Risk of bias assessment of the included RCTs. Figure S1: PRISMA flowchart Table S3. Definitions of composite kidney outcomes in the included randomised controlled trials (RCTs). Figure S3. Forest plot illustrating the effect of GLP‐1RAs on MACE after the exclusion of the FREEDOM‐CVO trial. Figure S4. Forest plot illustrating the effect of GLP‐1RAs on stroke after the exclusion of the SUSTAIN + PIONEER‐6 trial. [file DOM-27-6847-s001.docx]
